# Draft genome sequence of two *Aspergillus aculeatus* isolated from cashew nuts from coastal Kenya

**DOI:** 10.1128/mra.00460-24

**Published:** 2024-09-16

**Authors:** Manase Onyango Aloo, Pauline Wambui Gachanja, Eugene Mwanza Muzami, Kyalo Katua, Dennis Wamalabe Mukhebi, Colletah Rhoda Musangi, Bicko Steve Juma, Wilton Mwema Mbinda

**Affiliations:** 1 Department of Biochemistry and Biotechnology, Pwani University, Kilifi, Kenya; 2 Pwani University Bioscience Research Center (PUBReC), Pwani University, Kilifi, Kenya; 3 Department of Agroecology, Crop Genetics and Biotechnology Section, Aarhus University, Flakkebjerg, Denmark; University of Strathclyde, Glasgow, United Kingdom

**Keywords:** *Aspergillus aculeatus*, cashew nuts, Kenya, genome

## Abstract

*Aspergillus aculeatus* is a common saprophyte and ubiquitous fungus belonging to section *Nigri*. They produce diverse secondary metabolites which are important in biological processes and industrial applications. We present the draft genome sequences of two *A. aculeatus* isolated from cashew nuts from coastal Kenya.

## ANNOUNCEMENT

*Aspergillus aculeatus* is a filamentous fungus of the group black Aspergilli known for its production of secondary metabolites with a wide range of activities ranging from antifungal, antibacterial, antimicrobial, antifouling, and phytotoxic bioactivities (1). *A. aculeatus* grows in a wide range of substrates and under numerous conditions. However, it thrives well in tropical and subtropical regions where it abundantly inhabits soil and rotting fruits (2, 3). *A. aculeatus* produces several enzymes including hemicelluloses, amylases, and proteases which play an important role in food, feed, and other biotechnological processes (4). *A. aculeatus* is also a plant pathogen which causes leaf spot disease and rot diseases of fruits (5). It infests damaged fruits producing pectinase which breaks down the plant cell walls leading to the deterioration of fruits thus affecting their quality and shortening their shelf-life. Climate change is predicted to exacerbate the growth of these species and their impacts.

To understand its presence in cashew nuts and its impact on safety, we used pure *A. aculeatus* isolated from cashew nuts from coastal Kenya. Cashew nut samples were sampled in production areas (Kilifi, Kwale, and Lamu counties) of Kenya in May 2021 using a stratified random sampling approach (6, 7). After surface sterilization, cashew shells were cut into four sections and the kernels were cut into approximately 3 mm × 4 mm pieces. The particles were directly placed on modified rose bengal agar medium in Petri dishes and incubated at 30°C for 7 days in darkness. Fungi growing on the kernels were transferred to a water agar medium and incubated for 3 days at 27°C in the light. The resulting hyphae were further cultured on potato dextrose agar and malt extract agar media at 25°C for 7 days in the light to isolate pure cultures (8). Pure isolates were cultured at 25°C for 7 days in the dark for genomic DNA extraction using ZR Fungal/Bacterial DNA Miniprep kit (Zymo, Irvine, USA). Species identification was based on morphological determination and Sanger sequencing of PCR products of ITS (ITS1/4) and 28S rRNA regions (NL1/4) (9) and calmodulin gene (Cmd5/Cmd6) (10) at Macrogen, Netherlands. Genomic DNA from pure isolates was isolated from the mycelia using ZR Fungal/Bacterial DNA Miniprep kit (Zymo). The sequences were queried using NCBI BLASTn v2.14.0 (11). Sequencing library was generated using TruSeq DNA PCR-Free kit (Illumina, San Diego, USA) followed by whole genome sequencing using Illumina NovaSeq 6000, which generated 151 bp paired-end reads. The total number of reads for each genome is listed in Table 1. Default parameters were used for all software unless specified. Quality of the raw reads in fastq file formats was performed using FASTQC v0.12.1 (12) followed by trimming using fastp v3.3.5 (13) to remove the adapter sequences and low-quality bases. *De novo* filtered reads were assembled using SPAdes v3.15.5 (14). BUSCO v5.7.1 with the lineage database ascomycota_odb10 (15) was used to determine the completeness of the genome assemblies. The sequencing and assembly data are shown in Table 1.

**TABLE 1:** Sequencing and assembly data for *A. aculeatus* isolated from cashew nuts in coastal Kenya

Parameter	Isolate	
	2A	3B
Genbank accession no.	JBAPLX000000000	JBAPLW000000000
SRA accession no.	SRR28842120	SRR28842119
Number of reads	23,173,186	16,785,328
Genome size (bp)	36,366,186	35,721,297
Genome coverage (×)	94.5	69.7
% G + C	50.5	49.0
No. of contigs	128	195
N50 contigs (kb)	560.8	394.3
No. of scaffolds	50	100
N50 scaffolds (Mb)	2	1.1
Total no. of BUSCO orthologs	1,706	1,706
Complete single-copy, complete multicopy, fragmented, and missing orthologs (%)	98.4, 0.2, 0.3, 1.1	98.1, 0.1, 0.2, 1.6
ITS BLAST similarity (%), reference match, and accession	97, *Aspergillus niger*, MK108384.1	97, *Aspergillus aculeatus*, KP278205.1
28s rRNA BLAST similarity (%), reference match, and accession	99, *Aspergillus tubingensis*, MH866130.1	99, *Aspergillus niger*, MH872941.1
Calmodulin BLAST similarity (%), reference match, and accession	92.89, *Aspergillus aculeatus*, HM055489.1	96.38, *Aspergillus aculeatus*, LC573677.1

## Data Availability

The draft genome sequences have been deposited in GenBank under Bioproject number PRJNA1051575. The draft genome assemblies and raw sequencing reads were deposited with the accession numbers listed in Table 1.
